# Enhanced Oxidation Resistance of Polyphenylene Sulfide Composites Based on Montmorillonite Modified by Benzimidazolium Salt

**DOI:** 10.3390/polym10010083

**Published:** 2018-01-17

**Authors:** Jian Xing, Zhenzhen Xu, Bingyao Deng

**Affiliations:** 1 School of Textile and Garment, Anhui Polytechnic University, Wuhu 241000, China; xuzhenzhen@ahpu.edu.cn; 2 Key Laboratory of Science &Technology of Eco-Textile, Ministry of Education, Jiangnan University, Wuxi 214122, China; bydeng@jiangnan.edu.cn

**Keywords:** polyphenylene sulfide, organic montmorillonite, oxidation resistance, tensile properties, thermal stability

## Abstract

Organic montmorillonite (MMT) modified by 1,3-dihexadecyl-3*H*-benzimidazolium bromide (Bz) was used to prepare polyphenylene sulfide (PPS)/MMT composites by melting intercalation. The PPS/MMT composites showed mixed morphology, being comprised of exfoliated and intercalated structures with slight agglomerates. The tensile property of PPS/MMT composites was significantly improved due to the good dispersion of the MMT nanolayers. The test results showed that the tensile strength retention of PPS/MMT composites was higher than that of pure PPS after the oxidation treatment. Moreover, FTIR and XPS analyses were also used to evaluate the oxidation resistance of PPS composites. The FTIR analysis confirmed that adding MMT could better limit the damage of the C–S group and retard the generation of sulfuryl groups (–SO_2_–) during the oxidation treatment compared to pure PPS. The XPS analysis also suggested that the addition of MMT could reduce the chemical combination of the elements sulfur (S) and oxygen (O) during oxidation treatment. Furthermore, the MMT nanolayers could also promote the transfer of S from a C–S bond into an –SO_2_– group.

## 1. Introduction

Polyphenylene sulfide (PPS) is an attractive high-performance thermoplastic polymer which possesses high thermal stability, good mechanical properties and excellent fire resistance [1,2,3,4]. Due to its outstanding performance, PPS has been widely applied in the field of high temperature filtration [5,6]. However, PPS can be easily oxidized and crosslinked due to the low bond energy of the C–S–C bond. Therefore, research about improving the oxidation resistance of PPS has been reported over the last few decades [7,8,9]. Thus far, the methods of antioxidant modification can be mainly divided into two categories: (1) the surface coating method and (2) the direct addition of nanoparticles or antioxidants [6,8,9,10,11,12,13].

The direct addition of nanoparticles or antioxidants is commonly used to improve the oxidation resistance of PPS. Various nanoparticles such as silicon carbide (SiC), silicon dioxide (SiO_2_), montmorillonite (MMT), carbon black, etc. [11,13,14], have been directly added into PPS resin to improve the oxidation resistance. Sugama [9] used octadecylamine as a pretreatment agent to modify montmorillonite (MMT). The obtained organic MMT was then added to the PPS slurry coating. The results showed that the addition of MMT could retard the hydrothermal oxidation of PPS coatings. Zhang et al. [4] blended TiO_2_ and benzotriazole with PPS via melt compounding to prepare a PPS-based melt spun fiber. The tests showed that the addition of TiO_2_ and benzotriazole could significantly improve the light resistance of PPS fibers. The modified PPS melt spun fibers showed a weak color change after UV irradiation. Moreover, different kinds of antioxidants are also added into PPS resin by melt blending. Sugama [10] added three different antioxidants into PPS slurry coating, and this indicated that tetrakis [methylene (3,5-di-*t*-butyl-4-hydroxyhydrocinnamate)] methane (TMBHM) was most effective in hindering the hydrothermal oxidation of PPS coatings. In contrast with adding nanoparticles, the addition of antioxidants may create a wide range of problems, such as poor thermal stability of antioxidants, volatility of antioxidants during the melt processing, easy precipitation from the polymer matrix and low extraction resistance by the organic solvent. 

The surface coating method can also be used to improve the oxidative stability of PPS fiber products [15,16]. The antioxidants or nanoparticles are made into a conditioning fluid, which can form a protective coating on the surface of PPS fiber products by the impregnation or spray method. However, there are a lot of defects which limit its potential application, such as the nonuniformity of film, easy peeling of protective film, and high production costs. In conclusion, the direct addition method is a simple and effective way to improve the oxidation resistance of PPS.

Among those nanofillers used in the direct addition method, the MMT nanolayers have attracted intensive attention due to their excellent oxygen barrier properties. This is because the MMT nanolayers can prevent oxygen infiltration and efficiently delay the thermal oxidation of the polymer [17,18,19,20]. Nevertheless, the poor dispersion of MMT nanolayers in the polymer matrix can significantly influence the properties and application of polymer/MMT composites. For PPS/MMT composites, the MMT nanolayers can only present good dispersion in the PPS slurry coating [9]. The MMT nanolayers only show an agglomeration morphology in PPS resin which can be directly used to prepare fibers and nonwoven fabrics due to the poor thermal stability of organic MMT [21]. However, only a good dispersion of MMT nanolayers can be effective in preventing the transfer of oxygen and heat. Therefore, achieving good dispersion of MMT nanolayers in PPS resin using a benzimidazolium surfactant was studied in this work. The influence of MMT nanolayers on the oxidation resistance of PPS resin was also investigated. Moreover, the antioxidative mechanism of the MMT nanolayers on PPS resin was also reported in this work.

## 2. Experimental

### 2.1. Materials

PPS resin (melt flow index of 150 g/10 min at 315 °C/kg) was obtained from Ruitai Technology Co., Ltd. (Suzhou, China). A commercial Na–MMT (CEC = 92 mmol/100 g) was supplied by Fenghong Clay Co., Ltd. (Huzhou, China). Benzimidazole (98%), 1-bromohexadecane (98%), THF (99%) and NaOH (96%) were purchased from Sinopharm Chemical Reagent Co., Ltd. (Shanghai, China).

### 2.2. Preparation of Specimens

1,3-dihexadecyl-3*H*-benzimidazolium bromide (Bz) was used as an organic modifier to modify Na–MMT to obtain organic MMT (Bz–MMT), which aimed to improve the dispersibility and compatibility of MMT nanolayers in PPS resin. The synthesis of Bz and the modification of MMT had also been performed in our previous work [22]. 

A SISZ-10A corotate twin-screw extruder (Ruiming Co., Ltd., Wuhan, China) was used to manufacture PPS/Bz–MMT composites containing different contents of Bz–MMT (0.5, 1, 3, 5 and 10 wt %) by melt compounding. The melt compounding was continued for 8 min at a screw speed of 30 rpm at 290 °C. The PPS/Bz–MMT composites with various Bz–MMT contents were named as PPSBM*_x_*, where _x_ is the content of Bz–MMT. 

The specimens used for the tensile test were prepared by a SA-303 table-type test press machine (Tester Sanyo Co., Ltd., Osaka, Japan) and a SDL-100 sample cutting machine (Dumbbell Co., Ltd., Kobe, Japan). The PPSBM*_x_* composites were hot pressed at 295 °C under 20 MPa of pressure for 1 min, and then cooled down to room temperature under 20 MPa. Next, the obtained films were incised to make the specimens used for the tensile test. 

### 2.3. Characterization of Material Properties

The D-8 X-ray diffractometer (Bruker-axs Co., Ltd., Karlsruhe, Germany) with Cu Kα radiation (λ = 0.154 nm) at a generator voltage of 40 kV and a current of 40 mA was used to analyze the diffraction behavior of MMT and PPSBM*_x_* composites. Samples were pressed in stainless steel sample holders. All tests were conducted in the reflection mode at ambient temperature with 2θ varying between 1° and 20°. The scanning speed was 1°/min. The morphology and structure of PPSBM*_x_* composites were also characterized using a transmission electron microscope (TEM) H-9500 (Hitachi. Co., Ltd., Tokyo, Japan) with an accelerating voltage of 120 kV.

The tensile properties of PPSBM*_x_* composites were characterized by an EX-SX tensile tester (Tester Sangyo Co., Ltd., Osaka, Japan) with a 50 kN load cell. The gauge length of specimens was 12 mm, and the strain speed was 5 mm/min. The measurement of each sample was repeated at least 5 times. The thermal degradation behavior of PPSBM*_x_* composites was analyzed using a Q-500 thermogravimetric analyzer (TA Instruments Co., Ltd., New Castle, DE, USA) in a nitrogen atmosphere with temperature from 30 °C to 800 °C and with a heating rate of 10 °C/min.

The procedure of the oxidation resistance test was as follows. The specimens of PPSBM*_x_* composites were soaked in a mixed acid H_2_SO_4_/HNO_3_/HCl (1:1:1) solution at 90 °C for 48 h, which simulates the actual working environment of PPS resin [23]. After the acid exposure, the specimens were rinsed with distilled water five to seven times, and then the specimens were dried at room temperature. Lastly, the specimens were kept in a vacuum drying chamber for 24 h before the measurements were taken. The chemical group change of PPS was detected using the ATR-FTIR (Thermo Fisher Scientific Co., Ltd., Waltham, MA, USA). The test wave number range was from 4000 cm^−1^ to 400 cm^−1^. The elements analysis was performed using an AXIS-ULTRDLD multifunctional X-ray photoelectron spectrometer (XPS) (Shimadzu Co., Ltd., Kyoto, Japan).

## 3. Results and Discussion

### 3.1. Morphology of PPSBM_x_ Composites

**Figure 1:**
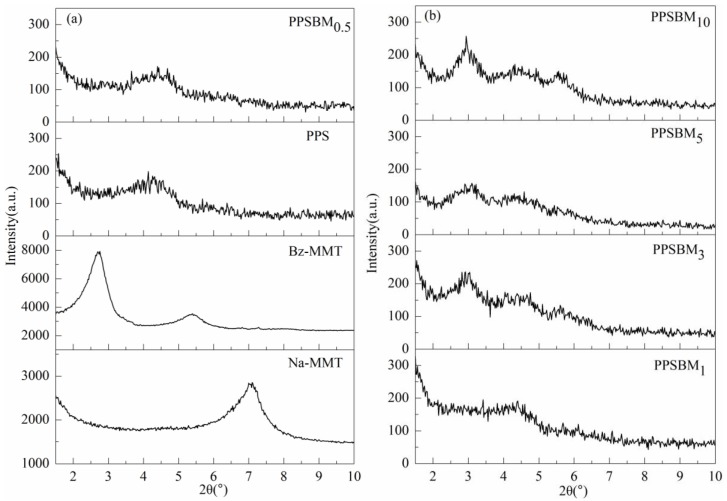
XRD patterns of pure polyphenylene sulfide (PPS) and PPSBM*_x_* composites: (**a**) Na–MMT, Bz–MMT, PPS and PPSBM_0.5_; (**b**) PPSBM_1_, PPSBM_3_, PPSBM_5_ and PPSBM_10_.

The dispersion and intercalated structures of Bz–MMT nanolayers in PPS matrix were characterized using XRD and TEM. The XRD patterns of Na–MMT, Bz–MMT, and PPSBM*_x_* composites are shown in Figure 1. The interlayer spacing of Bz–MMT in PPSBM*_x_* composites can be calculated using the Bragg equation based on the position of the diffraction peak. From Figure 1, it is shown that the diffraction peak of Na–MMT is 7.1° while the diffraction peak of Bz–MMT significantly shifts to a smaller angle and the diffraction angle reduces to 2.7°. Moreover, it can also be observed that no new diffraction peak appears except the diffraction peak of the PPS matrix when the content of Bz–MMT is less than 1 wt % for PPSBM*_x_* composites. However, when the load of Bz–MMT is more than 3 wt %, the appearance of two new diffraction peaks at 2θ = 3.0° and 5.7° can be evidently observed from Figure 1b. The new peaks are different from the diffraction peaks of Bz–MMT (2θ = 2.7° and 5.4°), but also the interlayer spacing is much larger than that of Na–MMT (1.2 nm) [22]. This phenomenon can be explained by the thermal degradation of Bz during the melt blinding and intercalation of PPS molecular chains. Therefore, from the XRD patterns, it can be calculated that the PPSBM*_x_* composites exhibit an exfoliated morphology when the content of Bz–MMT is low. However, the absence of the diffraction peaks in PPSBM*_x_* composites can also be attributed to low amounts of Bz–MMT, which are not easy to detect. The PPSBM*_x_* composites possess a mixed morphology of exfoliated and intercalated structures when the content of Bz–MMT is high. Therefore, the dispersion of Bz–MMT nanolayers in PPS resin cannot be estimated based only on the XRD patterns.

**Figure 2:**
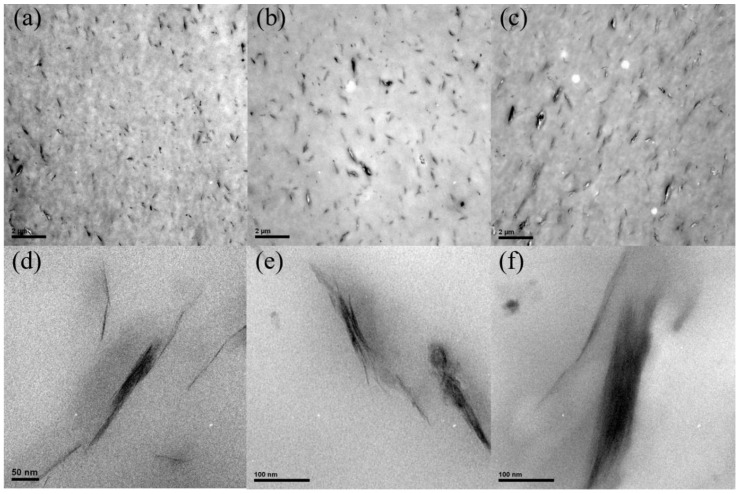
TEM images of PPSBM*_x_* composites: (**a**,**d**) 1 wt %, (**b**,**e**) 3 wt %, and (**c**,**f**) 5 wt %.

To determine the details of the Bz–MMT dispersion in the PPS matrix, the morphology of composites was also measured using TEM. Figure 2 shows the TEM photomicrographs of PPSBM*_x_* composites, which can be used to evaluate the previous analysis of XRD patterns. It is evident that Bz–MMT distributes well in the PPS matrix but still forms some agglomerates. From Figure 2a,d, it is evident that there are individual and intercalated nanolayers in the PPS matrix, as well as a few agglomerates. The individual and intercalated nanolayers are also observed in Figure 2b,e and Figure 2c,f, but the agglomerates of Bz–MMT nanolayers increase in number and size as the Bz–MMT content increases. Therefore, the observed dispersion of Bz–MMT from the TEM images is not consistent with the analysis of XRD patterns. Meanwhile, in comparison with our previous work, the Bz can improve the compatibility of Na–MMT and the PPS matrix. The particles of Bz–MMT can disperse, intercalate, and exfoliate under the shear stress during the melt blending process. Therefore, the PPSBM*_x_* composites exhibit a mixed morphology of exfoliated and intercalated morphology, but the agglomerates of Bz–MMT nanolayers cannot be avoided during the melt blending.

### 3.2. Tensile Properties of PPSBM_x_ Composites

**Figure 3:**
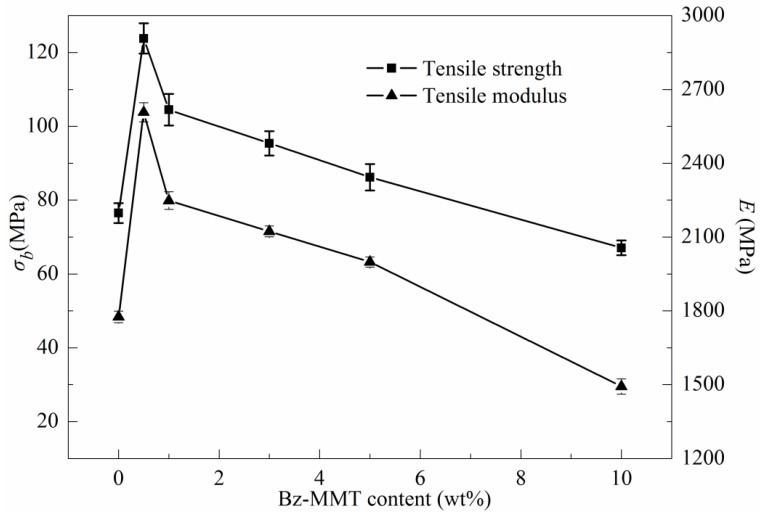
Tensile properties of PPS and PPSBM*_x_* composites: (σ*_b_*) tensile strength; (*E*) tensile modulus.

The tensile properties of pure PPS and PPSBM*_x_* composites are shown in Figure 3. It can be clearly observed that the tensile strength and tensile modulus of PPS resin significantly improves due to the addition of Bz–MMT. When the content of Bz–MMT is just 0.5 wt %, the tensile strength and tensile modulus increase from 76.5 MPa and 1775.8 MPa to 123.8 MPa and 2607.3 MPa, respectively. However, the tensile properties of PPSBM*_x_* composites decrease with increasing the Bz–MMT contents. When the content of Bz–MMT is up to 10 wt %, the tensile strength and tensile modulus are even lower than that of pure PPS. When a small amount of Bz–MMT is added into PPS resin, the Bz–MMT nanolayers can be exfoliated and intercalated in composites with slight agglomerations. The individual nanolayers can enlarge the propagation path of cracks and absorb energy to increase the plastic deformation during tensile stretching. Moreover, the interactions between nanolayers and PPS matrix can also significantly improve the tensile properties. The structure defects caused by a slight agglomeration of Bz–MMT nanolayers can be negligible. Therefore, the tensile properties of composites are improved. Furthermore, the MMT nanolayers can also act as heterogeneous nucleation agent to promote the crystallization of PPS, which can improve the tensile properties of PPS. However, with the increase of Bz–MMT content, the proportion of the exfoliated nanolayers decreases, and the proportion of the intercalated or agglomerated nanolayers increases. Additionally, the large agglomerates in Bz–MMT nanolayers can also cause serious structural defects, and the degradation of organic modifiers during melt blending can also induce the degradation of PPS macromolecular chains and cause the formation of structure defects in composites. Thus, the composites with a high content of Bz–MMT have a low tensile property.

### 3.3. Thermal Degradation of PPSBM_x_ Composites

**Figure 4:**
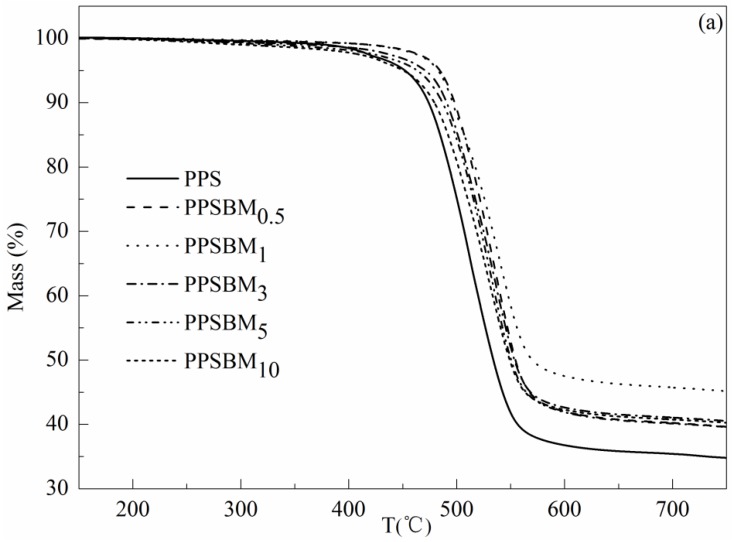

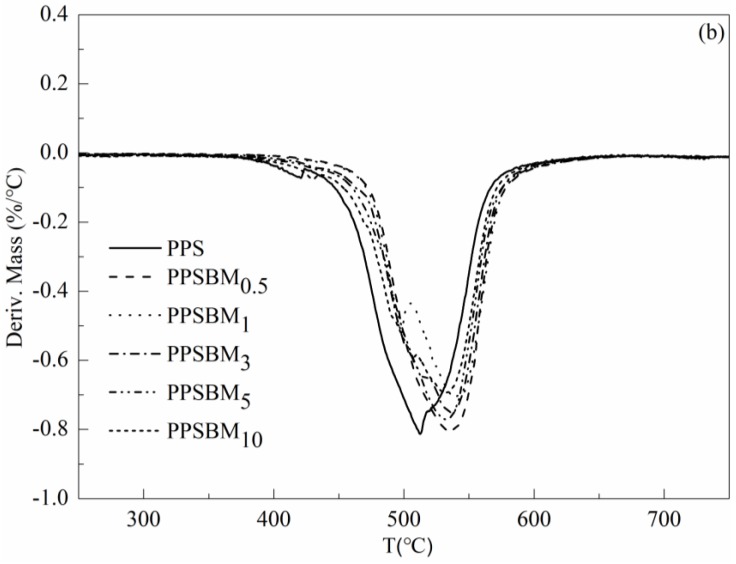
TGA (**a**) and DTG (**b**) curves of PPS and PPSBM*_x_* composites.

The TGA and derivative thermogravimetric analysis (DTG) curves of PPS and PPSBM*_x_* composites are plotted in Figure 4. Moreover, the degradation temperatures at different percentages of weight loss (*T*_5%_, *T*_15%_, *T*_30%_, and *T*_50%_) and the maximum decomposition rate (*T*_max_) are also listed in Table 1.

From Figure 4, it can be clearly observed that both pure PPS resin and PPSBM*_x_* composites exhibit a single stage thermal degradation with a large peak in DTG curves over the whole test temperature range. Furthermore, it is evident that the thermal stability of PPSBM*_x_* composites show a significant improvement compared with that of pure PPS resin due to the addition of Bz–MMT. From Table 1, it can be found that the *T*_5%_ of PPSBM*_x_* composites is much higher than pure PPS resin when the Bz–MMT content is less than 5 wt %. The *T*_5%_ of PPSBM_0.5_ is 484.5 °C, which is 32.3 °C higher than that of pure PPS. However, adding excessive Bz–MMT can also reduce the thermal stability of PPS due to the thermal degradation of the organic modifier at lower temperatures. The *T*_5%_ of PPSBM_10_ is 1.6 °C lower than that of pure PPS resin. The temperature corresponding to the *T*_max_ firstly increases but then decreases with the increase of Bz–MMT content. The enhancement in thermal stability of PPS composites can be attributed to the mass transport barrier effect of Bz–MMT nanolayers, which can restrict the diffusion of gaseous decomposition products and slow the heat transfer during degradation [24]. Moreover, the Bz–MMT nanolayers can also promote the PPS matrix to form protective carbon piles during the degradation process, which can delay or even stop the transfer of heat and gaseous decomposition products [25,26,27].

The heat-resistant index (*T_HRI_*) is also used to represent the thermal stability of polymers, and can express the temperature which limits the long-time use [28,29]. The *T_HRI_* can be calculated by the following equation:

*T_HRI_* = 0.49 × [T_5%_ + 0.6 × (*T*_30%_ − *T*_5%_)] (1)

where *T*_5%_ and *T*_30%_ is the corresponding degradation temperature of 5% and 30% weight loss, respectively. As shown in Table 1, the *T_HRI_* of composites also initially increases and then decreases with increasing additions of Bz–MMT. The *T_HRI_* of PPSBM_1_ is 13.8 °C higher than that of pure PPS. This can be explained by the mass transport barrier effect and favorable dispersion of Bz–MMT. However, adding a high content of Bz–MMT can agglomerate in the matrix and damage the overall structure of composites. Therefore, the thermal stability of PPSBM*_x_* composites decreases when the content of Bz–MMT is high. 

### 3.4. Oxidation Resistance of PPSBM_x_ Composites 

Under the conditions of high temperature or a strongly acidic environment, polymer molecular chains are susceptible to oxygen, which can attack and cause the rupture of macromolecular chains. Moreover, free radicals can be generated when the oxidative cleavage of chains take place. The chain reaction of free radicals can also further aggravate the oxidation degradation of polymer molecular chains, which will exacerbate the destruction of material structure, damage the polymer’s mechanical properties, and shorten the service life of the polymer.

At present, the methods of measuring the oxidation resistance of a polymer are varied. In this work, the changes of tensile properties, the type and content of functional groups, the element content and the valence state of the PPS composites are all used to characterize the oxidation resistance. This, in turn, can be used to explore the antioxidative mechanism of the Bz–MMT nanolayers on PPS resin.

#### 3.4.1. Tensile Strength Analysis

**Figure 5:**
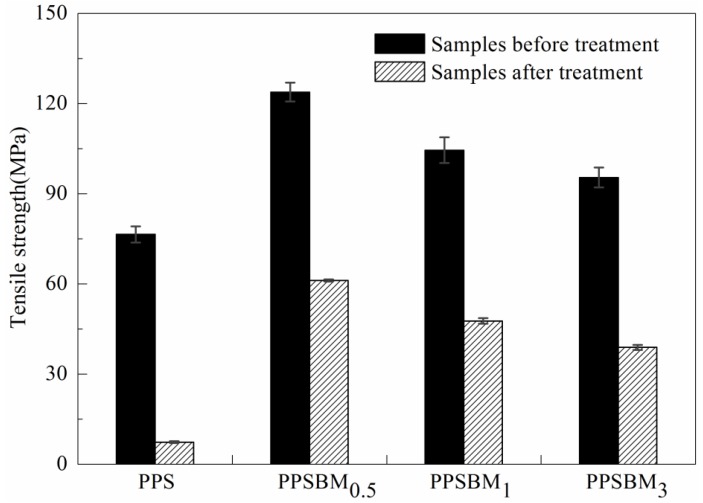
Tensile strength changes of PPS and PPSBM*_x_* composites after oxidation treatment.

Figure 5 shows the tensile strength changes of PPS and PPSBM*_x_* composites after oxidation treatment. It is found that the tensile strength of pure PPS resin reduced from 76.5 MPa to 7.4 MPa. The tensile strength loss ratio is as high as 90.3%. This indicates that the specimens are heavily oxidized by strong acid and high heat in the atmosphere of oxygen, which induces the oxidative cleavage of PPS molecular chains and a sharp decrease of tensile strength. In contrast, the tensile strength of PPSBM_0.5_ resin is reduced from 104.5 MPa to 47.6 MPa. The tensile strength loss ratio is only 54.3%. Moreover, the tensile strength of PPSBM_1_ resin is reduced from 95.4 MPa to 38.7 MPa. The tensile strength loss ratio is 59.4%. Therefore, the tensile strength loss ratios of PPSBM*_x_* composites are much smaller than that of pure PPS resin, and the tensile strengths of PPSBM*_x_* composites are also significantly higher than that of pure PPS resin. This phenomenon can be attributed to the good dispersion of the Bz–MMT nanolayers in PPS matrix, which can delay the diffusion of heat and O_2_, NO*_x_* and SO*_x_* in the PPS matrix due to the mass transport barrier effect. Moreover, the Bz–MMT nanolayers can also hinder the emission of oxidative degradation products in the PPS matrix. Furthermore, the Bz–MMT nanolayers can result in heterogeneous nucleation crystallization, which promote the crystallization and reduce the proportion of amorphous region of PPS matrix [22]. Therefore, the degradation number of PPS molecular chains decreases, and the oxidation resistance of PPSBM*_x_* composites increases compared to pure PPS resin.

#### 3.4.2. Chemical Functional Groups Analysis

**Figure 6:**
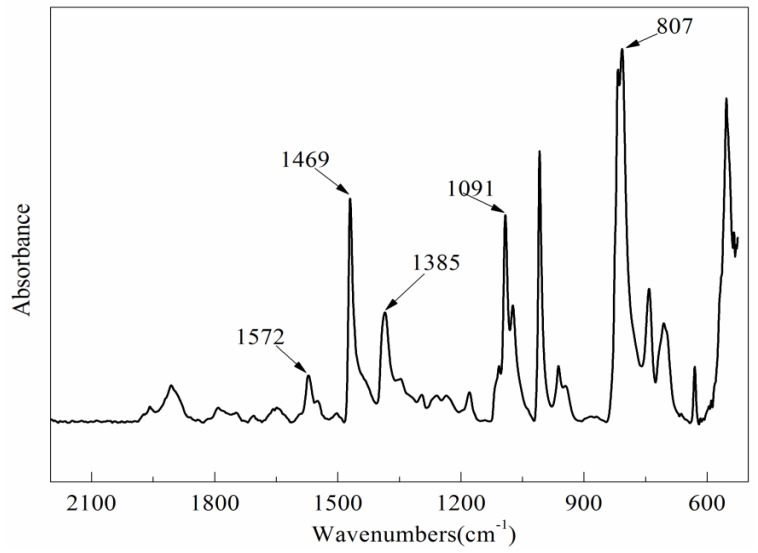
FTIR spectra of pure PPS resin.

The changes in chemical functional groups are measured using ATR-FTIR, which can be used to characterize the oxidation resistance and analyze the antioxidative mechanism. The FTIR spectrum of pure PPS resin is shown in Figure 6. 

It is found that 1572, 1469, 1385, 1091, and 807 cm^−1^ are the characteristic absorption peaks of pure PPS resin (Figure 6). The peaks at 1572, 1469, and 1385 cm^−1^ are attributed to the stretching vibration absorption of the benzene ring skeleton. The peak at 1091 cm^−1^ belongs to the stretching vibration absorption of the C–S bond. The strong absorption peak at 807 cm^−1^ is the characteristic peak of the ρ-disubstituted benzene ring. Moreover, the peak at 1178 cm^−1^ is attributed to the stretching vibration absorption of –SO_2_–, and 1075 cm^−1^ is the stretching vibration absorption of –SO– [3]. The existence of –SO_2_– and –SO– in pure PPS resin can be explained by the low bond energy of the C–S bond, which is easily oxidized during the synthesis, drying, and storage process of PPS resin. The peak at 1572 cm^−1^ also serves as a benchmark for comparison because it is the characteristic peak of PPS and cannot be attacked during oxidation treatment. Therefore, the relative absorbance of other absorption peaks can be obtained by comparison with its integral absorbance.

**Figure 7:**
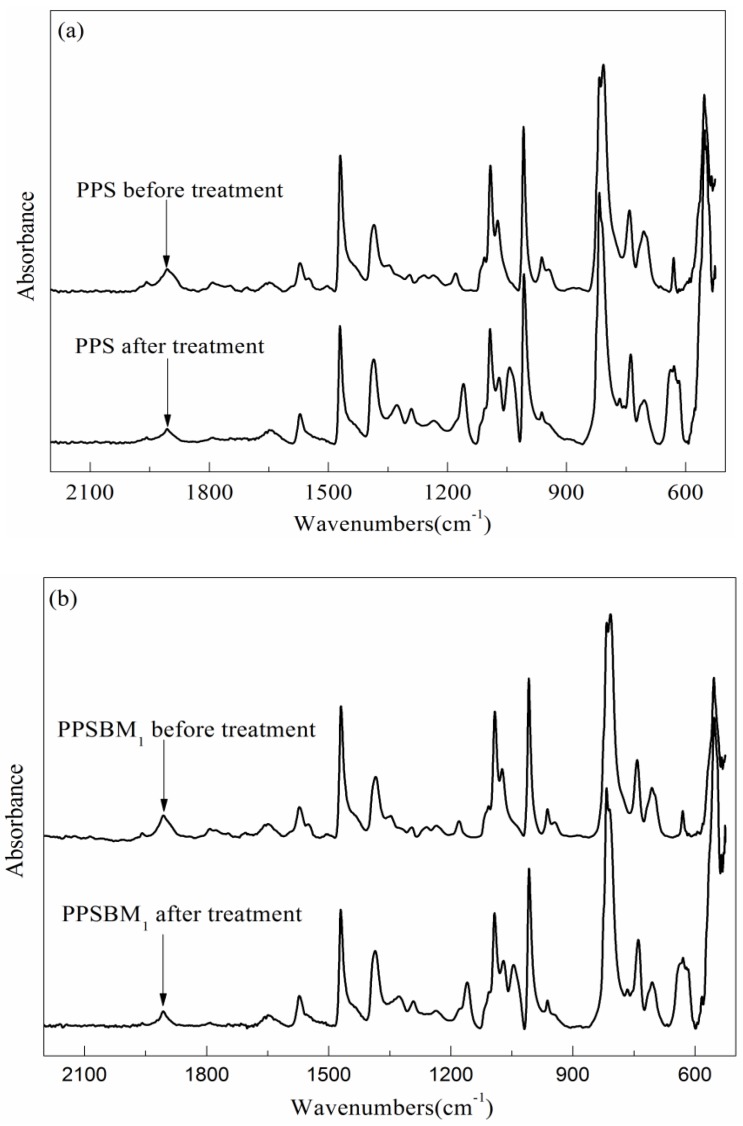
FTIR spectra of PPS (**a**) and PPSBM_1_ composites (**b**) after the oxidation treatment.

Figure 7 shows the FTIR spectra of PPS and PPSBM_1_ composites after the oxidation treatment. The relative absorbance of different chemical groups is also listed in Table 2. It can be found that there is no apparent change in the chemical groups of PPS, but a new absorption peak at 1044 cm^−1^ which can be attributed to aryl ether appears. This indicates that the PPS chains are oxidized to promote cross-linking during oxidation treatment. The relative absorbance of –SO_2_– at 1178 cm^−1^ has a significant increase, which indicates that the oxidation level of PPS resin intensifies. The relative absorbance of the C–S bond at 1091 cm^−1^ dramatically reduces, and the relative absorbance of –SO– at 1091 cm^−1^ also decreases. The reduction of –SO– can be explained by the fact that the bond order of S=O in –SO– is lower than that in –SO_2_–, which demonstrates that –SO– is more likely to be formed in PPS resin after the treatment. However, the S in –SO– has an unstable valence, and is easy to be oxidized again to form –SO_2_– under the condition of oxidation. Moreover, the relative absorbance of the ρ-disubstituted benzene ring at 807 cm^−1^ is also lower than that of PPS before the treatment, which suggests that the oxidative cross-linking takes place in PPS resin. This phenomenon is also consistent with the generation of the aryl ether. In short, the degree of oxidation in pure PPS resin intensifies, and the oxidative cleavage and cross-linking take place in PPS molecular chains after the treatment.

From Table 2, it can also be found that there is a big difference between the relative absorbance of pure PPS and PPSBM_1_ composites before oxidation treatment. This phenomenon can be explained by the fact that pure PPS resin and PPSBM_1_ composites are both blended in a twin-screw extruder for 10 min, and hot pressed by a table-type test press machine. Thus, the oxidation of PPS cannot be avoided in the process of melt blending or injection molding. It needs to be pointed out that adding Bz–MMT can delay the oxidation and cross-linking of PPS in the melting process. The relative absorbance of the C–S bond and the ρ-disubstituted benzene ring is clearly higher than that of pure PPS before treatment. In addition, the relative absorbance of –SO– and –SO_2_– is lower than that of pure PPS.

It is clear that the relative absorbance of the C–S bond in PPSBM_1_ composites is still higher than that of pure PPS. Additionally, its amount of decrease is lower than that of pure PPS after oxidation treatment, as seen in Figure 7 and Table 2. The amount of increase of –SO_2_– group in PPSBM_1_ composites is lower than that of pure PPS, but the amount of decrease of –SO– group in PPSBM_1_ composites is higher than that of pure PPS after oxidation treatment. The relative absorbance of the –SO_2_– group, –SO– group, and aryl ether in PPSBM_1_ composites are also lower than that of pure PPS after treatment. These phenomena indicate that the addition of Bz–MMT delays oxidation and cross-linking during the oxidation treatment. There are two possible reasons for this. Firstly, the Bz–MMT nanolayers can form the exfoliated structure or intercalated structure in the PPS matrix. Thus, the nanolayers can limit the diffusion of O_2_ and acid in the PPS matrix due to mass transport barrier effect. The nanolayers can also restrict the transfer of oxidative degradation products. Secondly, the Bz–MMT nanolayers can promote the crystallization and perfect the crystal structure [22], which hinders the entrance of O_2_ and acid into PPS crystals. Therefore, the addition of Bz–MMT improves the oxidation resistance of PPS resin.

#### 3.4.3. Surface Elements Analysis

**Figure 8:**
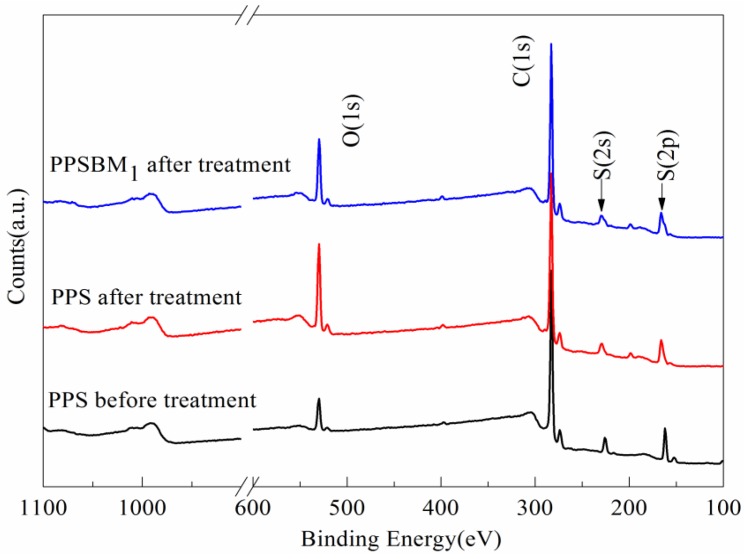
XPS spectra of PPS and PPSBM_1_ composites after treatment.

To gain a better understanding of the antioxidative mechanism of Bz–MMT on PPS, the element content and the valence state of PPS and PPSBM_1_ composites are also measured by an XPS. The XPS spectra of PPS and PPSBM_1_ composites after normalized processing are shown in Figure 8. It can be found that PPS and PPSBM_1_ composites mainly contain the elements oxygen (O), carbon (C), and sulfur (S) from Figure 8 [30]. After the oxidation treatment, the relative intensity of the O in pure PPS and PPSBM_1_ composites significantly increases, but the amount of increase in pure PPS is much higher than that in PPSBM_1_ composites. The relative intensity ratio of O and C in pure PPS resin is 0.27 before oxidation treatment. After the treatment, it reaches 0.54 for pure PPS, and only 0.42 for PPSBM_1_ composites. This confirms that S and C in PPS combine with a large amount of oxygen during the oxidation treatment, while the addition of Bz–MMT can limit this combination of oxygen to improve the oxidation resistance of PPS.

**Figure 9:**
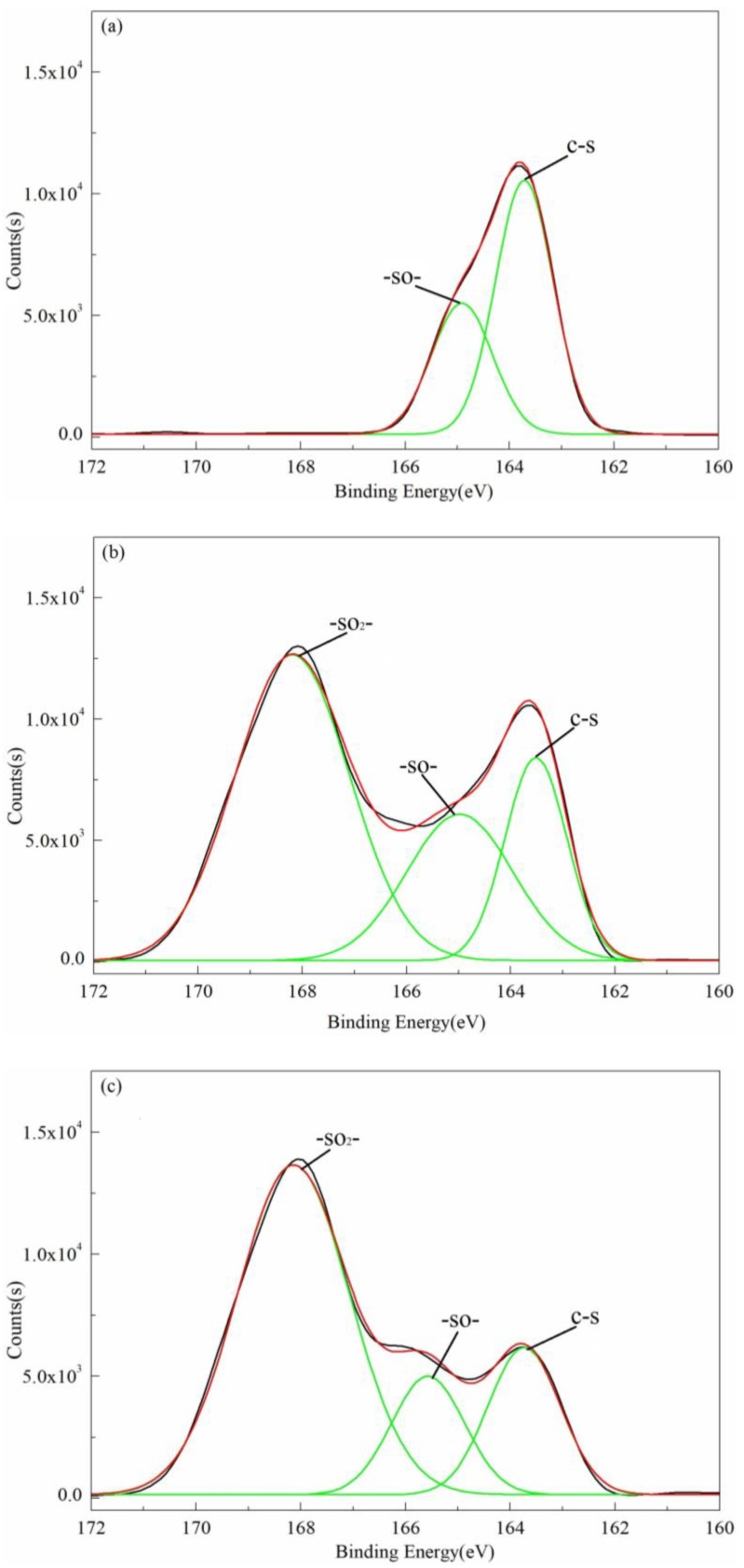
XPS spectra of S_2p_ in the pure PPS and PPSBM_1_ composites: (**a**) pure PPS before treatment, (**b**) pure PPS after treatment, (**c**) PPSBM_1_ composites after treatment.

To further research the valence state changes of the S element, a high-resolution scan of S_2p_ is also performed. Figure 9 shows the fitting curves of the S_2p_ XPS spectra of PPS and PPSBM_1_ composites. The relative contents of different functional groups of S are also listed in Table 3. From Figure 9a, it is evident that the S_2p_ peak of pure PPS before the treatment can be deconvoluted into two major peaks at 163.7 eV and 164.9 eV. These are attributed to the C–S bond and –SO– group, respectively [30,31,32]. It can also be observed that the element S in PPS is mainly found in the C–S bond from Table 3. Moreover, a proportion of the element S (32.6%) exists in the form of –SO–, because the element S can be easily oxidized during the process of storage and melting manufacture. 

The S_2p_ peak of pure PPS after the treatment can be deconvoluted into three peaks at 163.7 eV, 164.9 eV, and 168.2 eV. These are attributed to the C–S bond, –SO– group, and –SO_2_– group, respectively [30,31,32], as observed from Figure 7b. It is clear that the proportion of the C–S bond significantly decreases, while the proportion of the –SO_2_– group greatly increases after oxidation treatment. The relative content of the C–S bond decreases from 67.4% to 14.1%, and that of –SO_2_– group can reach 56.3%. This confirms that significant oxidation has taken place in pure PPS resin. For PPSBM_1_ composites after the oxidation treatment, the S_2p_ peak can also be deconvoluted into three peaks corresponding to the C–S bond, –SO– group, and –SO_2_– group, respectively. From Table 3, it can also be found that the content of various functional groups is similar to that of pure PPS after the oxidation treatment. However, the relative content of the C–S bond is higher than that of pure PPS, which indicates that the addition of Bz–MMT can prevent the oxidation cleavage of the C–S bond in PPS molecular chains. 

It is interesting to note that pure PPS has a higher relative content of the –SO– group than PPSBM_1_ composites after the oxidation treatment. The relative content ratio of the –SO– group and –SO_2_– group is 1:1.9 for pure PPS, while that of PPSBM_1_ composites can reach 1:4.3. This suggests that the content of the –SO_2_– group is richer in PPSBM_1_ composites after the oxidation treatment. This phenomenon is also consistent with the above ATR-FTIR analysis. Therefore, the addition of Bz–MMT can promote the transfer of the element S from the C–S bond to the –SO_2_– group in PPS macromolecular chains, which have a structure similar to polyarylene sulfide sulfone (PASS). The S in the –SO_2_– group reaches the stable valence state and can remain stable under the condition of oxidation. Therefore, the surface of PPS can form a proactive layer with a PASS-like structure due to the addition of Bz–MMT, and this improves oxidation resistance.

## 4. Conclusions

The addition of Bz–MMT can significantly improve the tensile strength of PPS. However, adding a high content of Bz–MMT can also reduce the tensile properties of PPS due to the agglomeration and degradation of Bz–MMT. The thermal stability of PPS is also remarkably improved by the mass transport barrier effect of Bz–MMT nanolayers. However, excessive Bz–MMT also reduces the thermal stability of PPS because of the degradation of the organic modifier. The oxidation resistance analyses confirm that the addition of Bz–MMT markedly decreases the tensile strength loss ratio of PPS after the oxidation treatment. Moreover, adding Bz–MMT can also delay the oxidation cleavage of the C–S bond and reduce the generation of –SO– groups, –SO_2_– groups, and aryl ethers. It also restricts the joining together of S in PPS macromolecules and O from the external environment. The addition of Bz–MMT promotes the transfer of S from the C–S bond to the –SO_2_– group in PPS macromolecular chains. Overall, the antioxidant mechanism of Bz–MMT nanolayers on PPS resin has two explanations. Firstly, the barrier effect of Bz–MMT nanolayers can limit the diffusion and transfer of heat, oxidizing substances, and oxidation products in the PPS matrix in order to retard the oxidation rate. Secondly, adding Bz–MMT can promote the formation of –SO_2_– group in the PPS macromolecule chains, which can form a protective layer with a PASS-like structure on the surface of PPS to improve oxidation resistance.

## Figures and Tables

**Table 1:** TGA parameters of PPS and PPSBM*_x_* composites.

Samples	*T*_5%_ (°C)	*T*_15%_ (°C)	*T*_30%_ (°C)	*T*_50%_ (°C)	*T_HRI_* (°C)	*T*_max_ (°C)
PPS	452.2	484.7	507.0	533.9	237.7	512.3
PPSBM_0.5_	484.5	508.9	528.1	554.4	250.2	536.5
PPSBM_1_	482.9	507.1	533.5	570.1	251.5	537.8
PPSBM_3_	470.9	501.1	524.8	553.6	246.6	536.6
PPSBM_5_	463.5	498.7	522.6	550.6	244.5	532.5
PPSBM_10_	450.6	492.1	518.4	549.1	240.7	532.1

**Table 2:** Relative absorbance of PPS and PPSBM_1_ composites before and after the treatment.

Wavenumber (cm^−1^)	Relative Absorbance			
	PPS before Treatment	PPS after Treatment	PPSBM_1_ before Treatment	PPSBM_1_ after Treatment
1572	1.00	1.00	1.00	1.00
1178	0.31	1.97	0.27	1.76
1091	3.62	2.52	3.71	2.89
1075	1.32	0.79	0.99	0.16
1044	-	2.46	-	2.40
807	9.92	9.27	11.07	9.57

**Table 3:** Comparison of different functional group in S_2p_ for PPS and PPSBM_1_ composites.

Samples	Relative Contents (%)		
	C–S	–SO–	–SO_2_–
PPS before treatment	67.4	32.6	-
PPS after treatment	14.1	29.6	56.3
PPSBM_1_ after treatment	18.8	15.6	65.6
